# Mixture Detection Using a Deep-UV Raman-LIBS Autofocus-Based Compact Chemical Spectroscopic Sensor

**DOI:** 10.1021/acsomega.5c03801

**Published:** 2025-07-14

**Authors:** Atchutananda Surampudi, Anil Aryal, Tilak Hewagama, Narasimha Prasad, Dina M. Bower, Mool C. Gupta

**Affiliations:** † Charles L. Brown Department of Electrical and Computer Engineering, 744402 University of Virginia (UVA), Charlottesville, Virginia 22904, United States; ‡ Laser and Plasma Technologies (LPT), LLC, Charlottesville, Virginia 22904, United States; § 53523 NASA Goddard Space Flight Center, Greenbelt, Maryland 20771, United States; ∥ 53524 NASA Langley Research Center, Hampton, Virginia, 23666, United States; ⊥ Department of Astronomy, College Park MD, 1068 University of Maryland, College Park, Maryland 20742, United States

## Abstract

We present a compact, multifunctional chemical sensor that seamlessly integrates deep-UV Raman and laser-induced breakdown spectroscopy (LIBS) modalities into a single lightweight hand-held unit. By employing a single 266 nm laser source (1.5 ns pulse width, 10 mW average power) and an integrated autofocus mechanism, this design overcomes the complexities associated with systems that rely on dual or multiple laser wavelengths (e.g., 532 or 1064 nm). The 3D-printed sensor body weighs only 38 g and occupies a compact volume of 70 × 60 × 40 mm^3^ (that can fit within a palm of a hand) enabling comfortable hand-held operation in both laboratory and field environments. When combined with a 215 g deep-UV compact laser unit and a 90 g compact but high-resolution spectrometer (which is possible only with deep-UV operation), the overall system weight remains under 500 g, reinforcing its suitability for highly mobile applications. The functionality of the sensor is demonstrated for mixture detection in (a) a complex mineral-planetary simulant mixture, (b) isotope mixture, and (c) an organic–inorganic mixture. The deep-UV 266 nm operation allowed mixture detection to as low as 0.1% with such a compact sensor, which is only possible with bulky intensified CCDs previously reported with visible/IR wavelengths. The deep-UV excitation enhances Raman signal strength and reduces fluorescence interference, while the integrated autofocus capability facilitates seamless switching between LIBS and Raman operation modes. Compared to existing integrated approaches, this single-laser design significantly reduces optical complexity and overall system footprint, offering a robust solution for in situ chemical analyses ranging from environmental monitoring to planetary exploration.

## Graphical abstract

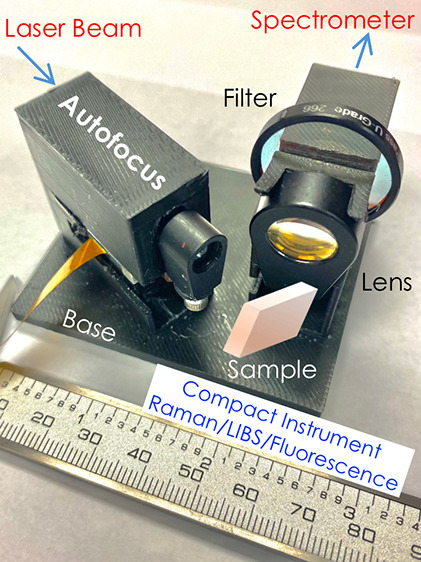

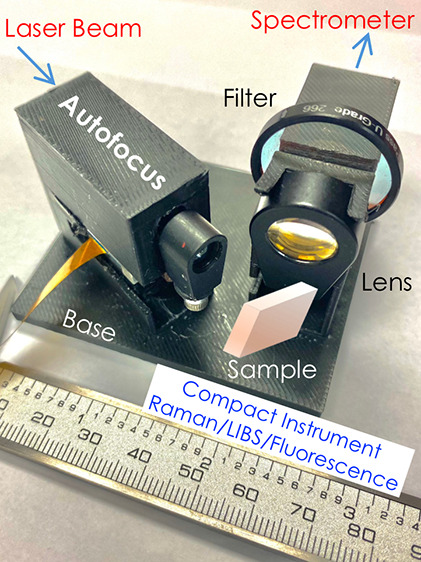

## 1 Introduction

Optical spectroscopic sensing techniques have evolved into indispensable tools for material characterization across a broad range of applications, from environmental monitoring and food authentication to planetary exploration.^1–3^ Among these, Raman spectroscopy and laser-induced breakdown spectroscopy (LIBS) have emerged as complementary techniques.^4,5^ Raman spectroscopy provides molecular fingerprinting by detecting vibrational modes, whereas LIBS delivers rapid elemental composition analysis through laser-induced plasma emissions. Recently, the integration of these modalities into a single platform has drawn considerable attention, as evidenced by several studies that have successfully combined LIBS and Raman techniques to address complex analytical challenges.^6–11^

For instance, Vaisakh et al. (2023) in^6^ demonstrated an integrated LIBS-Raman approach to monitor microplastics and heavy metal contamination in water resources. Their work underscored the synergy between the two techniques, highlighting how molecular and elemental data can provide a comprehensive understanding of environmental samples. Similarly, Shin et al. (2023) in^7^ applied a hybrid Raman and LIBS system for food authentication, exploiting the complementary nature of the spectral signatures to detect adulteration in food products. Another noteworthy contribution is by Clegg et al. (2014) in,^8^ who explored the use of these combined techniques for geochemical analysis under Venus-like atmospheric conditions. Their study not only illustrated the robustness of integrated spectroscopic approaches under extreme conditions but also set the stage for future applications in planetary exploration. In a more recent effort, Pinson et al. (2023) in^9^ integrated LIBS and Raman spectroscopy with machine learning techniques for interrogating weatherization in lithium hydride, providing an example of how advanced data analysis can augment the capabilities of spectroscopic systems.

Although these studies have demonstrated significant progress, most integrated systems rely on dual or multiple laser sources, for example, employing 532 or 1064 nm for Raman and LIBS, respectively. For LIBS measurements, the sample needs to be ablated, which means a pulsed laser is employed. However, for Raman measurements, a low-power laser continuous wave laser is employed, also preferably at a different wavelength. This multilaser approach, while effective, poses several challenges. It necessitates complex optical layouts involving separate beam paths, intricate alignment procedures, and multiple calibration steps. Moreover, the increased number of optical components can lead to a bulkier and costlier system, which is not ideal for field deployment or space-constrained applications. The necessity for dual wavelength systems also raises issues regarding the optimization of laser parameters, as each modality has different excitation requirements that can compromise performance when combined.

In contrast, this work presents a compact sensor, offering a novel solution by employing a single deep-UV laser operating at 266 nm for both Raman and LIBS measurements. Instead of using multiple wavelengths, the sensor employs an autofocus mechanism, wherein, using a single laser, the beam is either focused or defocused to allow or prevent ablation and seamlessly obtain LIBS/Raman signatures from samples under consideration. For the autofocus mechanism, the positions of the autofocus to prevent ablation are carefully considered via analytical calculations. The deep-UV regime provides intrinsic advantages over visible or near-infrared wavelengths, such as enhanced Raman scattering efficiency and reduced fluorescence interference (compared to visible/IR wavelengths). These advantages translate into more robust spectral signatures and allow for the sensitive detection of target materials even at low concentrations. Furthermore, the use of a deep-UV wavelength allows the use of a compact spectrometer that allows high spectral resolution with high UV sensitivity. Importantly, integration of an autofocus mechanism in our system enables rapid and precise switching between LIBS and Raman modalities by merely adjusting the focal position. This single-laser strategy significantly simplifies the optical design while maintaining, or even enhancing, performance.

Our sensor’s compactness is another critical feature that sets it apart. By utilizing a 3D-printed integrated design, the sensor body weighs only 38 g and occupies a volume of 70 × 60 × 40 mm^3^, making it truly hand-held and easily deployable in challenging environments. When combined with a compact 215 g laser unit and a 90 g spectrometer, the entire system weighs less than 500 g. This represents a remarkable reduction in both size and weight compared to traditional systems, enabling a broader range of field and in situ applications.

The motivation behind this work is manifold. For planetary exploration, there is a pressing need for lightweight, multifunctional sensors that can perform comprehensive analyses with minimal payload. Traditional systems like SHERLOC, SuperCam,^10–13^ have employed deep-UV laser 248.6 nm for Raman measurements, and 520 nm for LIBS measurements. These often involve intricate optical assemblies and are limited by their reliance on multiple laser sources.^14,15^ Similarly, terrestrial applications such as environmental monitoring and food quality assurance demand rapid, reliable, and portable instrumentation that can provide both molecular and elemental insights in real time. By consolidating these capabilities into a single instrument, our design aims to offer a versatile and cost-effective alternative that does not compromise on performance.

In this manuscript, we detail the design, construction, and performance evaluation of our compact deep-UV Raman-LIBS sensor. We demonstrate its effectiveness through a series of experiments involving mineral mixtures, isotopic water mixtures (D_2_O/DI water), and organic–inorganic mixtures (IPA/DI water), achieving detection limits down to 0.1 vol %. The experimental results clearly show that our single-laser approach not only simplifies the sensor architecture but also enhances operational efficiency and sensitivity. Furthermore, we compare our findings with recent literature to highlight the practical advantages and potential applications of our sensor.

In summary, by merging the strengths of deep-UV Raman and LIBS in a single, lightweight package, this work contributes a significant advancement to the field of spectroscopic chemical sensing. The sensor is well-suited for applications that demand high performance in environments where size, weight, and simplicity are paramount, from remote field studies to planetary missions.

## 2 The Setup of the Optical Sensor

The schematic diagram of the setup is shown in Figure 1a. The yellow box depicts the arrangement of the components of the compact sensor. The laser (Crylink, 266 nm, 1.5 ns pulse width, 10 mW average power) and the spectrometer (Avantes Avaspec 4096 CL 250–355 nm) are external to the sensor, which nonetheless are compact and lightweight, and can be placed in a bag-pack. The output of the laser is coupled into the sensor as the beam is collimated via a collimator (Edmund 88–173) and is focused using a lens L1 (Thorlabs LA4280, *F* = 10 mm), onto the sample. The lens L1 sits on an autofocus (AF) translation stage/actuator (Newscale M3L, range = 6000 μm). Off-axis to the laser delivery, the scattered beam from the sample is collimated using a lens L2 (Thorlabs LA4647, *F* = 20 mm). The collimation from lens L2 is needed to allow the light to be filtered through a high-pass edge filter EF (Semrock LP02–266RU-25, edge wavelength 268.6 nm). This filtering is needed to reduce the transmission of Raleigh scattered stray light from the sample. The filtered light is focused by a lens L3 (Edmund 88–181). The light is focused into a fiber (Avantes UVVIS 600 μm) through which the light reaches the spectrometer.

**Figure 1:**
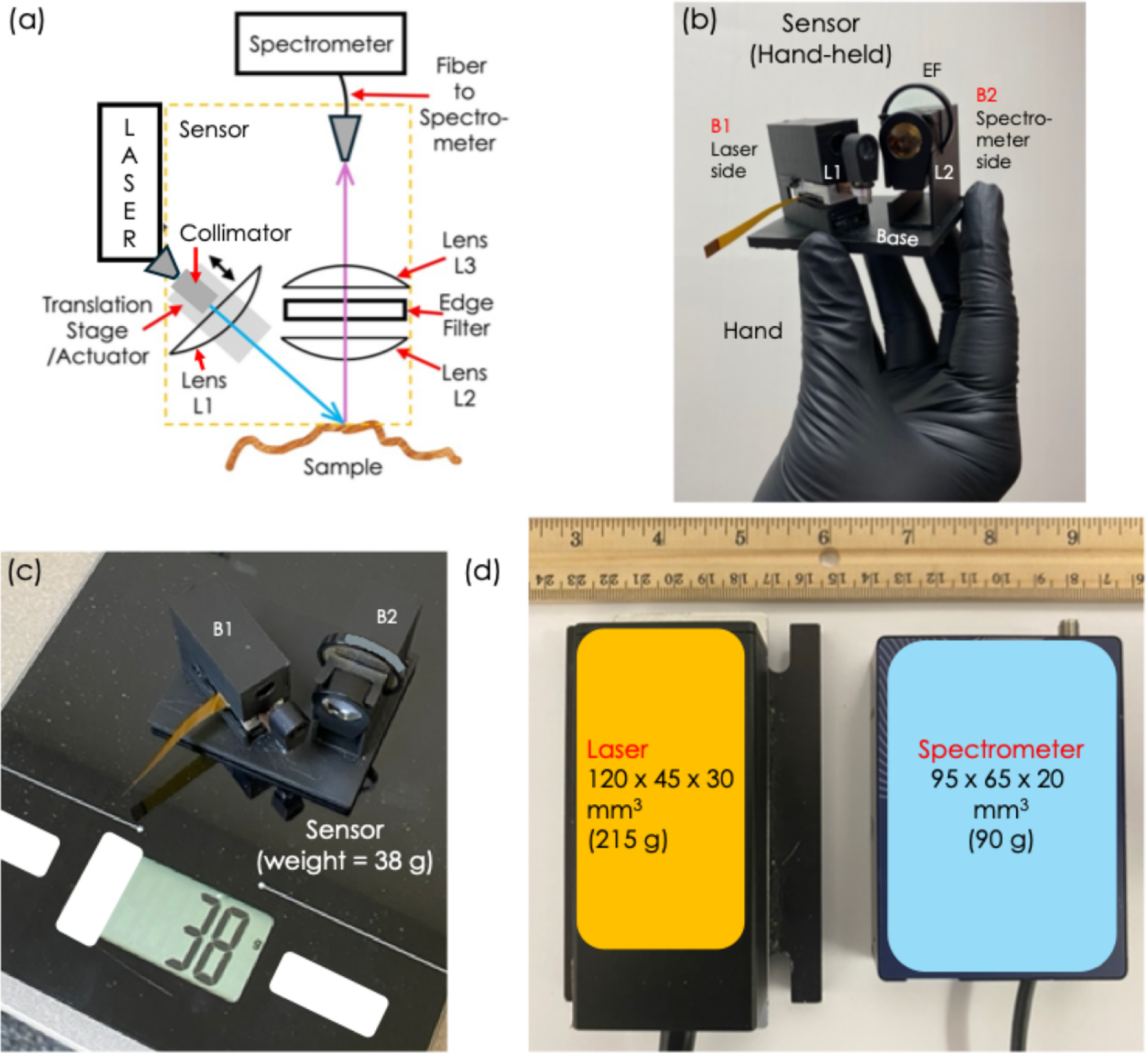
(a) The schematic diagram of the setup, (b) The hand-held sensor, (c) the weight measurement of the sensor = 38 g, (d) the size comparison of the entire setup with the laser and the spectrometer.

In the assembly of the sensor, the components including the collimator, the autofocus actuator (AF), and the lens L1 are housed into a compact body unit B1. The body unit B1 represents the laser-side components. The lens L2, edge filter (EF), and the lens L3 are housed into a compact body unit B2. This body unit B2 represents the spectrometer-side components. The body units B1 and B2 are 3D printed as a single integrated unit on a base of thickness 3 mm, as shown. The entire assembly of the sensor (B1, B2 and base) becomes a compact unit which can be hand-held, as shown in Figure 1b. As shown in Figure 1c, the sensor weighs just 38 g, which is significantly lightweight compared to the weight any multifunctional sensor previously reported (>1000 g). The sensor is placed in proximity to the laser and the spectrometer, as shown in Figure 1c. The laser occupies compact volume of 120 × 45 × 30 mm^3^ with a weight of 215 g (additional power supply unit: 168 × 88 × 140 mm^3^, weighing 200 g); and the spectrometer occupies a compact volume of 95 × 65 × 20 mm^3^ with a weight of 90 g. The sensor occupies a volume of 70 × 60 × 40 mm^3^, with a weight of 38 g, as mentioned. So, overall, all three components combined would not weigh more than 500 g. The laser and the spectrometer can be carried as a portable compact bag-pack unit (just weighing ∼300 g), and the sensor can be hand-held, allowing portable detection.

The sensor was 3D printed based on the CAD design as shown in Figure 2. The following discussion will allow one to CAD design the sensor independently in the future. Figure 2a shows the front-view of the sensor, and Figure 2b shows the top-view of the sensor. The scale is 10 mm per grid square. It should be noted from the top-view in Figure 2b, that the long axis of the body unit B1 is at an angle of 60° to the long axis of body unit B2. The distance between the home position of the lens L1 on the autofocus stage to the sample is 6000 μm. Body unit B2 has a groove to accommodate the edge filter EF. The front perspective view is shown in Figure 2c. The autofocus stage AF is accommodated by inserting into the slot AF in body unit B1. As shown in the back perspective view in Figure 2d, the body units B1 and B2 have grooves (10 mm in diameter) to accommodate the fibers, the collimator, and lens L3 that deliver/collect the laser/scattered light.

**Figure 2:**
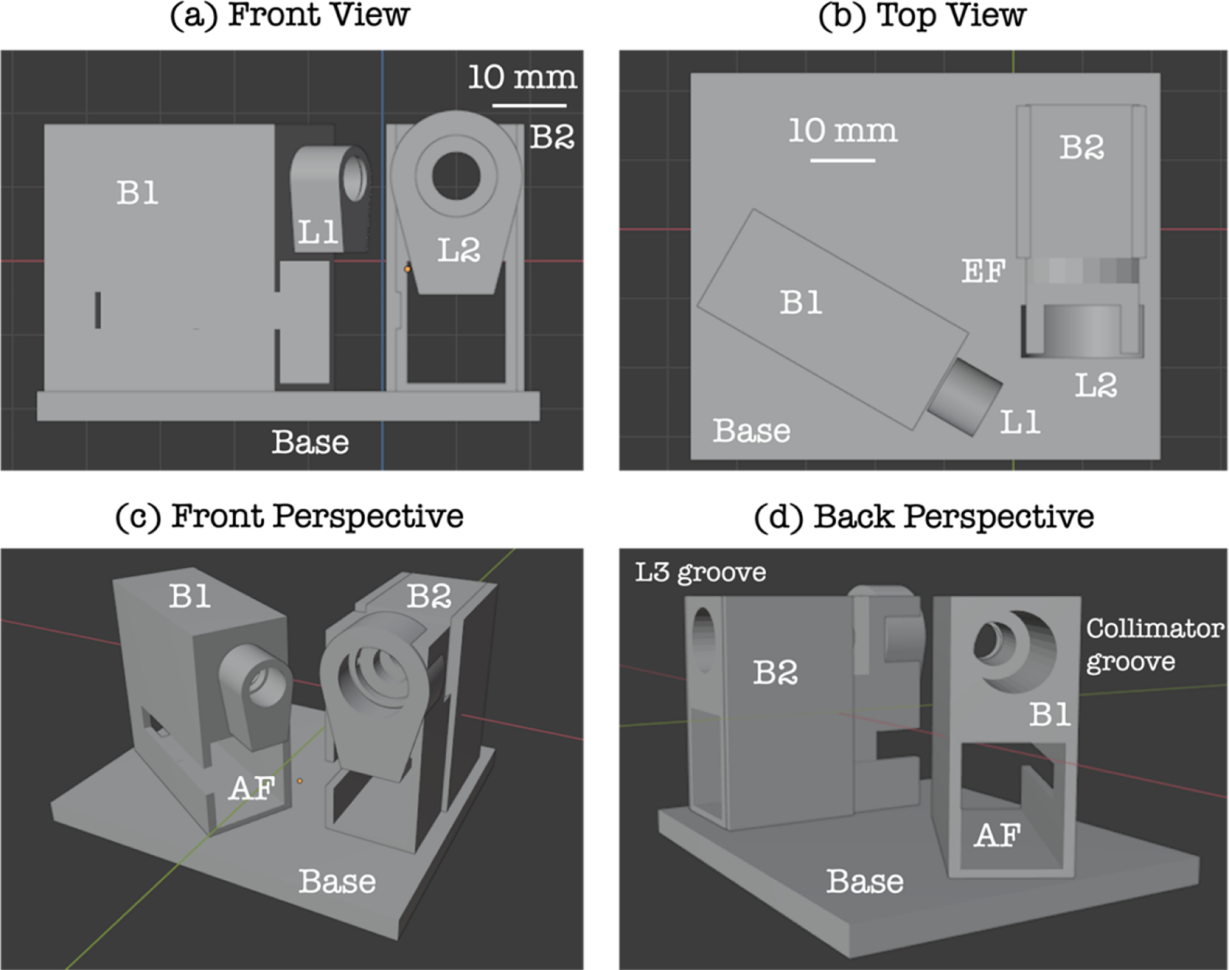
(a) Front view, (b) top view, (c) front perspective view, (d) back perspective view.

### 2.1 Autofocus Capability

The lens L1 is placed on the autofocus stage in order to allow focusing and defocusing of the input laser beam onto the sample, just by translating the lens L1. For the LIBS measurements, the lens L1 would be set at the position so that the beam is focused and ablates the sample. Based on the design of the sensor, the 3000 μm position from home position of the lens L1, is determined as the focus position, imparting maximum fluence. For Raman measurements, the L1 is translated to defocus from the focus position such that the fluence is below the ablation threshold. At focus, for a laser pulse energy of 10 μJ, and with the NA = 0.22 of the lens L1, a fluence of ∼1.27 J/cm^2^, is imparted on a sample, which is above the ablation threshold for minerals and simulants with 266 nm.^16^ For the Raman measurements, to prevent ablation, the beam should be defocused, and the fluence should be < 0.1 J/cm^2^,^17^ while still ensuring enough photons for Raman detection. To calculate the corresponding translation of the lens L1, the hyperbolic trajectory of beam propagation near focus is considered, as shown in Figure 3. Here, as the beam propagates along + *z* axis, the trajectory of the beam could be expressed as shown in eq (1), where *x* is the radius of the beam; and *a* and *b* are constants of the hyperbola, as expressed in eq (2), for NA being the numerical aperture of the lens.

**Figure 3:**
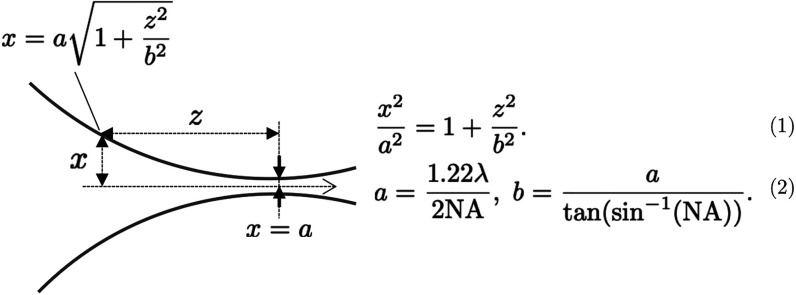
Hyperbolic trajectory of beam along + *z* direction,and the evolution of the radius of the beam along *x* direction.

Based on these equations in (1) and (2), and inserting the value of NA of lens L1 = 0.22, and wavelength of 266 nm, we calculate *a* = 1.48 μm, and b = 6.54 μm. At focus, *z = 0* and *x = a*, and the fluence was obtained ∼1.27 J/cm^2^. To reduce the fluence to <0.1 J/cm^2^, which is by at least 13 times, the radius of the beam should be increased by ∼4 times. From eq (1), substituting *x = 4a*, we get, z = 6.2 μm. The lens L1 is defocused by at least ∼30 μm for Raman measurements.

Based on these analytical considerations, for Raman measurements, to prevent ablation, the lens L1 is moved by ∼± 50 μm from the focus position of 3000 μm (used for LIBS). A distance of movement of ±50 μm is a compact short distance, allowing a compact design of the sensor. This autofocus capability of the sensor allows it to seamlessly switch between the choice of measurements (Raman or LIBS). This adds to the novelty of the sensor because unlike the conventional Raman-LIBS setups where two lasers are used (a low-power continuous wave laser for Raman and a pulsed laser for LIBS), only a single laser is needed, which further allows for compactness.

## 3 Results and Discussion

The first demonstrations of mixture detection using the sensor were made over three kinds of matrix mixtures—(a) a mineral–planetary simulant mixture of Olivine (sourced from Ward’s Science geology collection in Rochester, New York) and W30 (a Pyroxene-based powder sourced from NASA Goddard Space Flight Center); (b) an isotope mixture of deuterium oxide (D_2_O) (sourced from Sigma-Aldrich) in deionized (DI) water, (c) an organic–inorganic mixture of isopropyl alcohol (IPA) (sourced from Sigma-Aldrich) in DI water. These three types of mixtures represent a useful combination of different geological and organic/inorganic matrices sufficiently allowing the testing of the functionality of the sensor. It is to be noted that the prepared mixture samples (b,c) in the following experiments were frozen (<− 5 °C) below the melting point of the sample. However, when the measurements were made, the laser beam caused a localized melting of the mixture sample to the liquid state. Therefore, the measurements are considered to be performed in the liquid state of the mixtures at the location of laser incidence, which will be the case even in cold geological environments due to the high energy of the laser.

### 3.1 Mineral - Planetary Simulant Mixture

Three samples were prepared as follows. Samples 1 and 2: fine grained powders of (1) W30, and (2) Olivine were separately spread on different glass substrates and pressed into pellets. Sample 3: a 50:50 wt % ratio of both powders was uniformly mixed, then spread on a third glass substrate, and pressed into a pellet. Microscope images of all three samples using a high magnification microscope (Hirox RH 8800) were obtained with a 4000× magnification. These are shown in Figure 4. From Figure 4a,b, W30 and relatively darker Olivine grains of ∼3–5 μm in size can be observed. From Figure 4c, the mixture sample shows both light and dark grains, confirming the preparation of a uniform mixture. To further confirm the mixture preparation, X-ray diffraction (XRD) results were obtained using Rigaku Smart Lab X-ray diffractometer (5.0° Selection & Soller slit, PSA open, length limiting slit = 10 mm). The XRD results are shown in Figure 5. The 2θ peaks of (a) pure W30 are marked in black, and (b) pure Olivine are marked in red. Particularly, for Olivine, the 2θ peaks at 52.54° and 62.66° are distinct in comparison with the W30 peaks. In the mixture sample, as shown in Figure 5c, the distinct peaks of both W30 and Olivine are clearly visible. This confirms the preparation of a uniform mixture.

**Figure 4:**
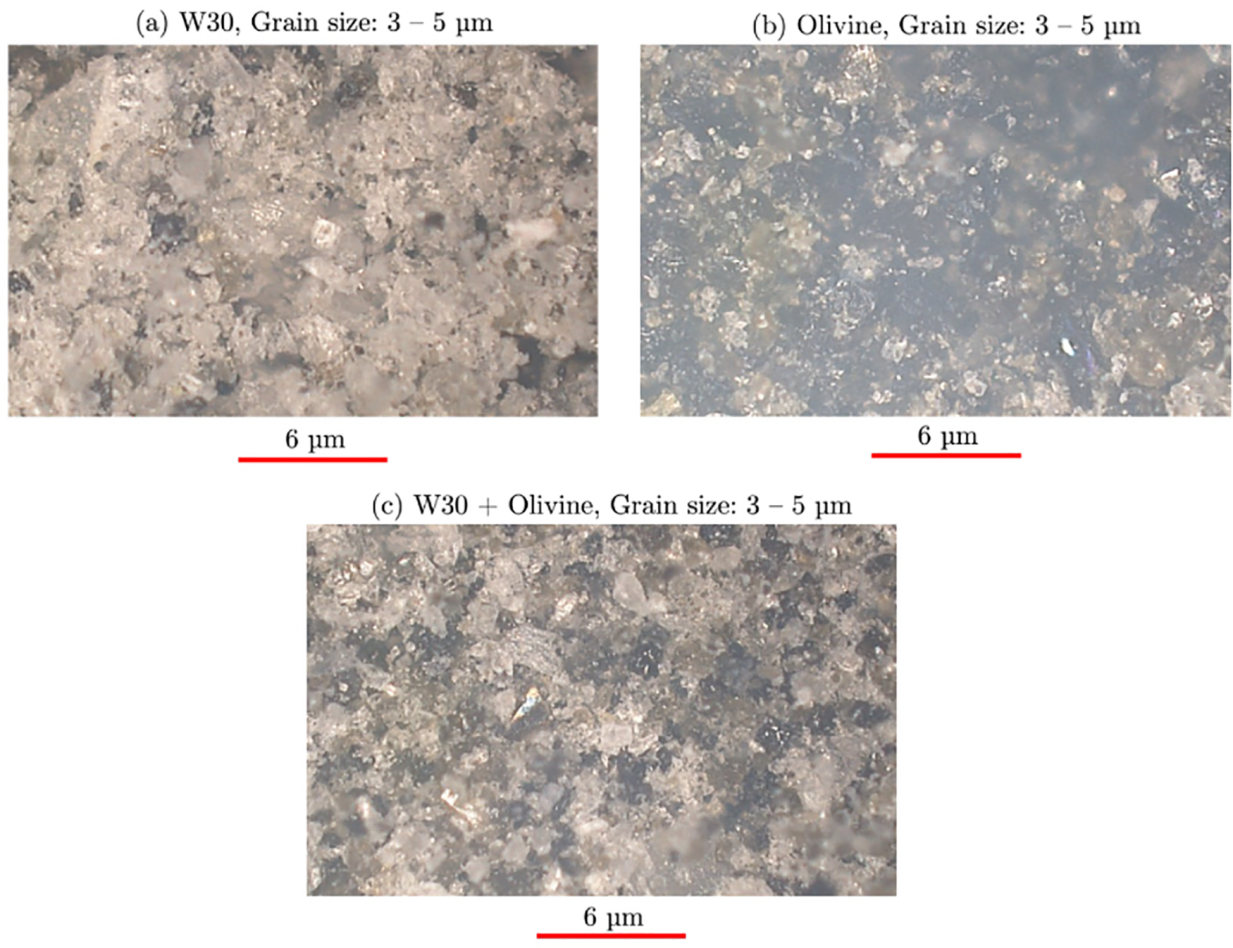
Microscope images: (a) sample 1: W30, (b) sample 2: olivine, (c) sample 3: mixture of olivine and W30.

**Figure 5:**
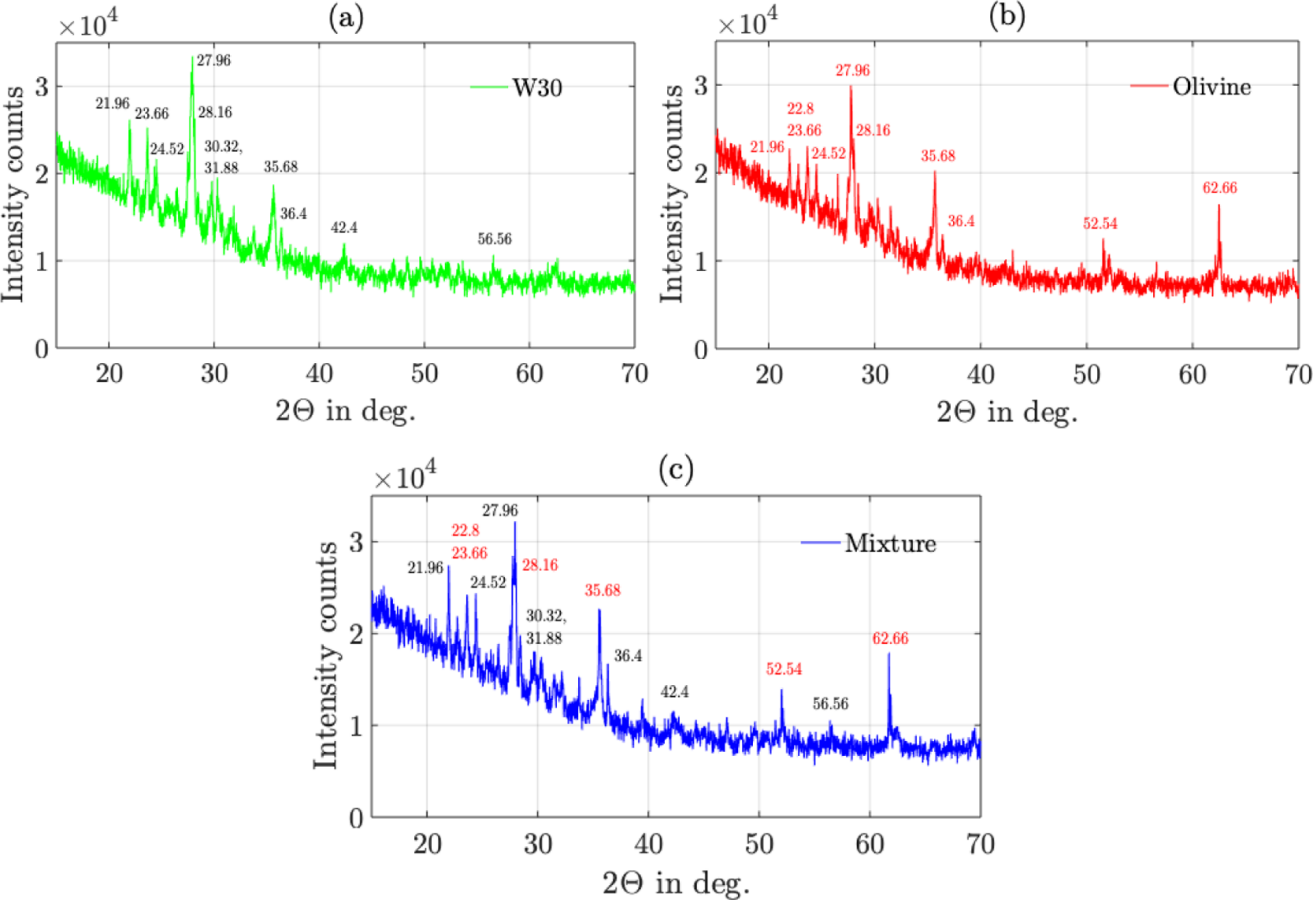
XRD results: (a) sample 1: W30, (b) sample 2: olivine, (c) sample 3: mixture of W30 and olivine.

The Raman results are then obtained for the samples using the sensor. The results are shown in Figure 6. From Figure 6a, a peak at 615 cm^–1^ is observed for the pure W30 sample, which indicates the presence of Pyroxene, as expected for the W30 simulant. From Figure 6b, twin peaks at 822 cm^–1^ and 856 cm^–1^ are observed, as expected for the pure sample of Olivine. For the mixture sample, in Figure 6c, the sensor detects the peaks of both W30 and Olivine in the mixture. This experiment is repeated for the sensor at different locations on the sample slides, and uniform results are obtained within an error of ±50 intensity counts. This highlights the ability of the sensor to perform mixture detection using its Raman functionality.

**Figure 6:**
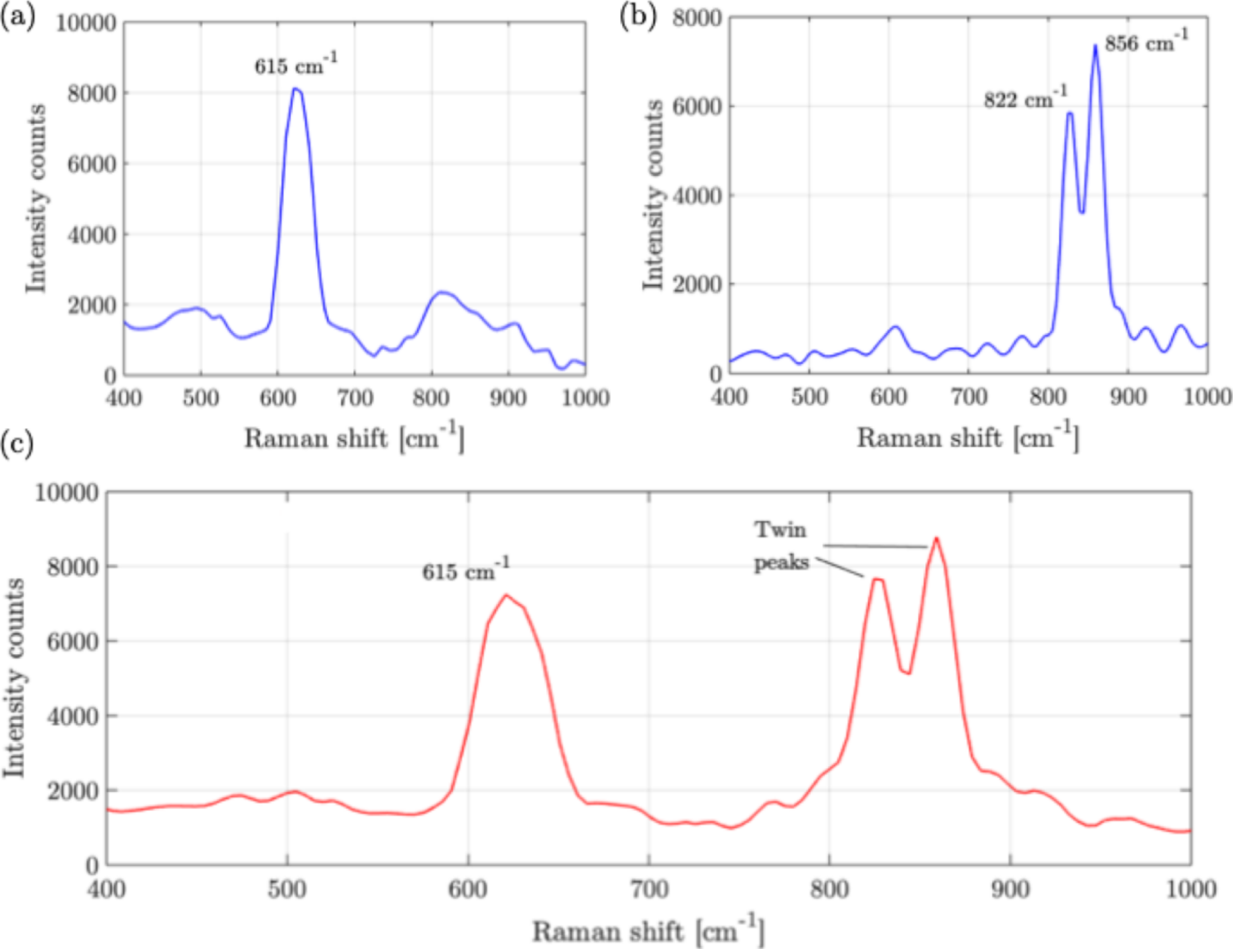
Raman results: (a) sample 1: W30, (b) sample 2: olivine, (c) sample 3: mixture of W30 and olivine. Slit size: 200 μm × 1 mm, integration time: 30s, averaging: 10 times.

The LIBS results were then obtained using the sensor for all the three samples. From Figure 7a, for pure W30 sample, strong LIBS peaks are detected for constituent elements of Fe, Mg, Mn, Si, Al, Ca, and Ti. From Figure 7b, for pure Olivine sample, the sensor is able to detect strong LIBS peak for Mg and Si. In the mixture sample, in Figure 7c, the sensor is able to detect strong peaks dictated by both W30 and Olivine—corresponding to Mg and Si from Olivine and Al from W30. This further highlights the ability of the sensor to detect mixtures using the LIBS detection functionality.

**Figure 7:**
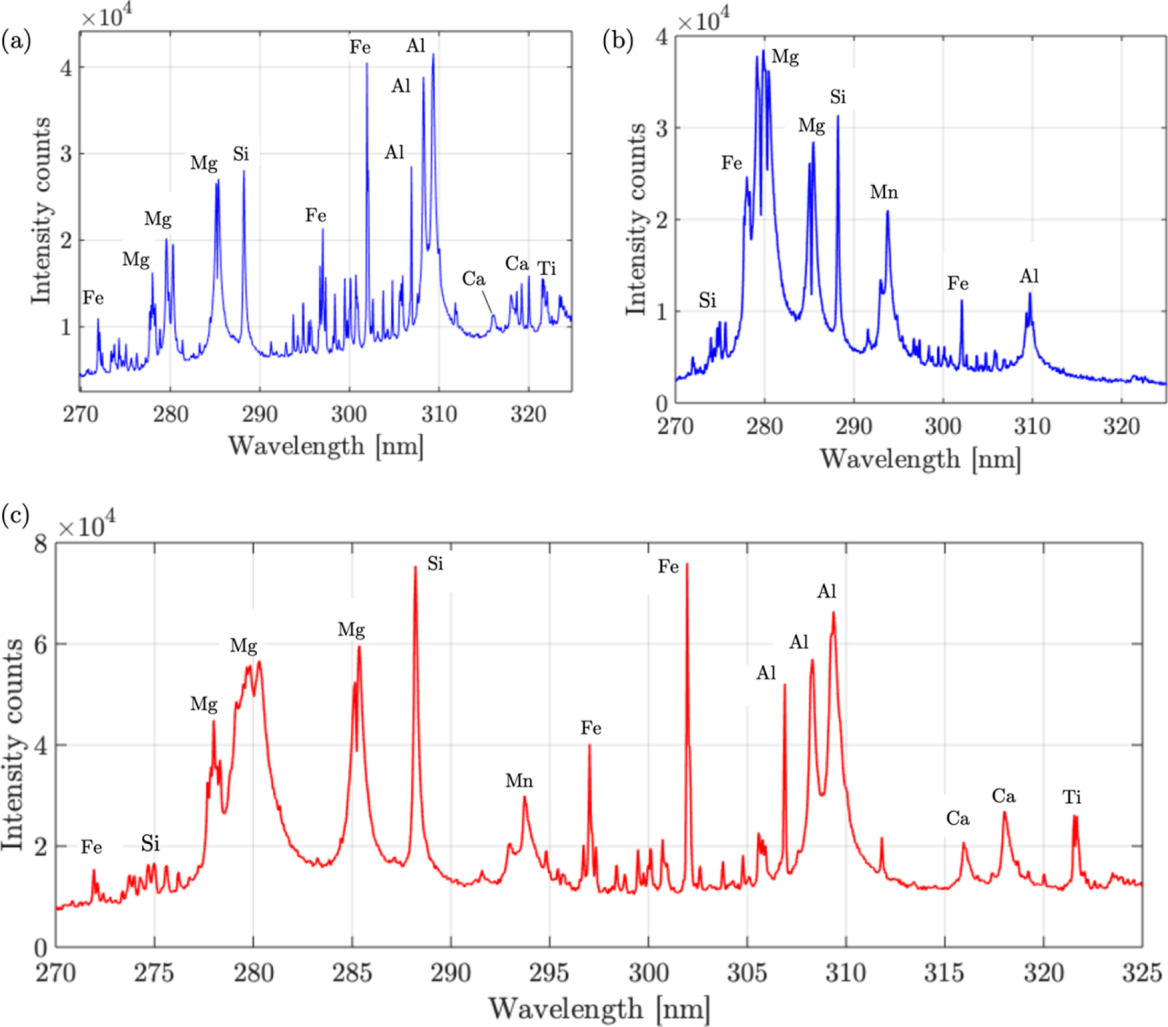
LIBS results: (a) sample 1: W30, (b) sample 2: olivine, (c) sample 3: mixture of W30 and olivine. Slit size: 200 μm × 1 mm, integration time: 30s, averaging: 10 times.

Additional measurements were made on the samples to highlight the potential of the sensor to detect mixtures using fluorescence as well. This measurement was made alongside Raman measurement while the lens L1 was moved to the defocused position in the Raman mode. Since the spectrometer used in the system is limited in the spectral range up to 355 nm, the fluorescence measurements were made using a different spectrometer Stellarnet Silver Nova VIS-IR (only for the purpose of highlighting the fluorescence detection capabilities of the sensor). The results are shown in Figure 8. The sensor is able to detect fluorescence emitted by pure W30 sample at 655 nm, and from pure Olivine sample at 715 nm. For the mixture sample, the sensor is able to detect an envelope spectrum where both the peaks from W30 and Olivine are detected. This further highlights the ability of the sensor to detect mixtures using the fluorescence detection functionality. One could note that to obtain Raman, fluorescence, and LIBS measurements at the same location, the Raman and fluorescence measurements were first carried out, and then the LIBS data were obtained to avoid changes in material composition due to the ablation process. The measurements are not simultaneous, but they allow for information about the material to be obtained using all three techniques at the exact location.

**Figure 8:**
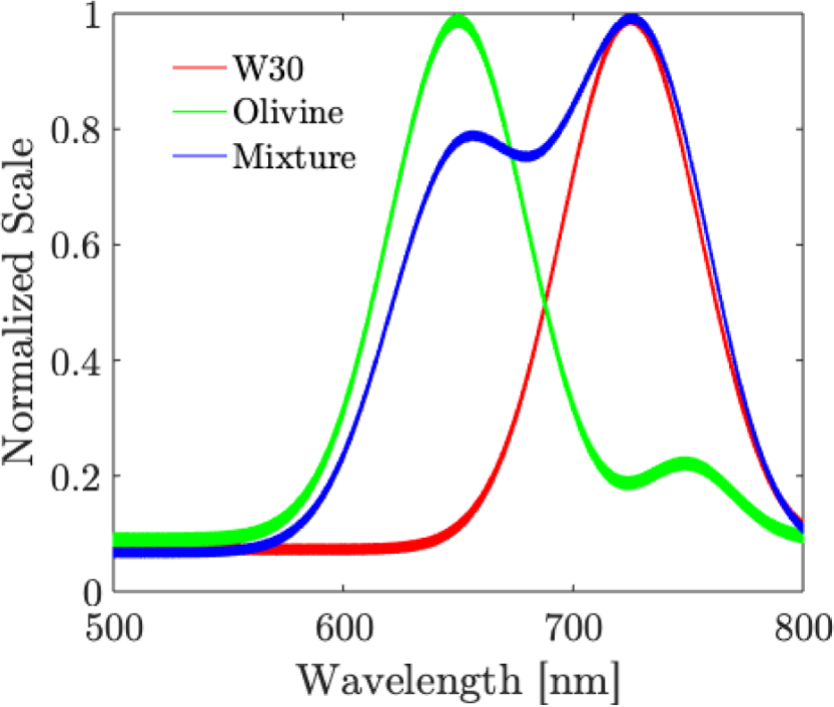
Fluorescence measurements with the sensor. Slit size: 200 μm × 1 mm, integration time: 30s, averaging: 10 times. (b) Isotope mixture.

### 3.2 Isotope Mixture

Different volumes % of D_2_O in DI water, i.e., 50%, 30%, 10%, 1%, and 0.1%, were prepared and filled in UV quartz cuvettes and frozen below the melting temperature. To allow higher sensitivity, the Raman measurements were initially made using the sensor with the largest available slit size of 200 μm × 1 mm at the spectrometer. The measurements were made over an integration time of 30 s, each averaged over 10 times. The results are shown in Figure 9.

**Figure 9:**
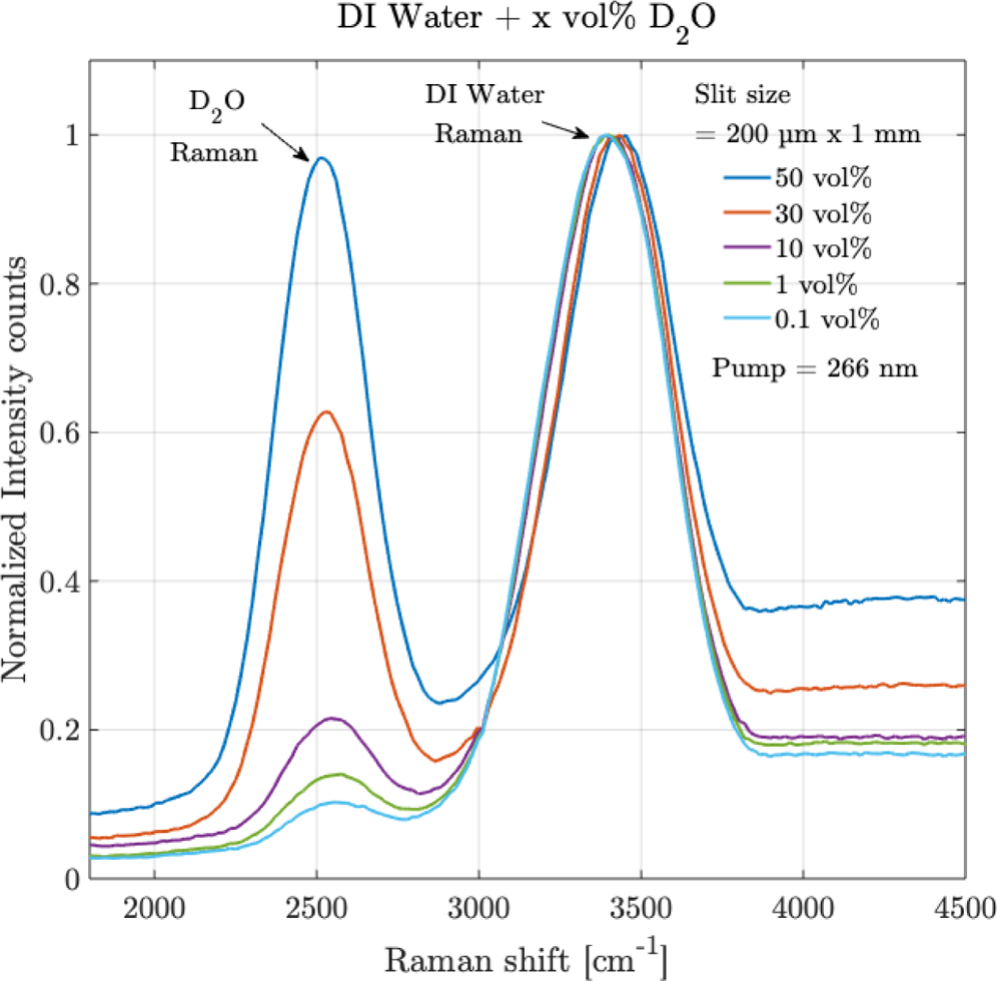
Raman measurements are shown for the isotope mixture of deuterium oxide (D_2_O) in DI water over different volumes % of D_2_O, of 0.1%, 1%, 10%, 30%, and 50%. The *Y*-axis intensity counts are normalized concerning the Raman peak of DI water. Slit size: 200 μm × 1 mm, integration time: 30s, averaging: 10 times.

It is observed in Figure 9, at 50 vol % of D_2_O, the Raman peaks of D_2_O and DI water are distinguishable, respectively occurring at 2508 cm^–1^ and 3421 cm^–1^, having a separation of 913 cm^–1^ as expected. As the volume % reduces, the sensor can still detect the Raman peak of the isotope up to as low as 0.1 vol %.

This detection of isotopes even at 0.1 vol % mixture is possible due to three reasons—(a) the use of a deep-UV laser providing a higher Raman scattering from the isotope material compared to a visible laser in conventionally used Raman instruments; (b) a high quantum efficiency of 84% at 266 nm deep-UV wavelength of the spectrometer; and (c) a wide Raman shift of 913 cm^–1^ between the isotopes which enabled the use of a large slit size (of 200 μm × 1 mm) leading to sensitive detection without any inter-Raman spectral interference, despite the cost of a low resolution (20 cm^–1^).

At this juncture, an additional experiment was conducted to observe if a smaller slit size, 50 μm × 1 mm, providing a higher resolution of 7 cm^–1^, could be useful to detect the Raman peak of the isotope as well. The comparative results are shown in Figure 10. As expected with a larger slit size, since a larger slit size allows more light to pass into the spectrometer, the Raman peak of D_2_O at 0.1 vol % is more intense and clearer with the 200 μm × 1 mm slit than with the 50 μm × 1 mm slit, therefore highlighting the benefit of a larger slit size. Nonetheless, the Raman peak of the isotope can still be detected by the sensor with a 50 μm slit use.

**Figure 10:**
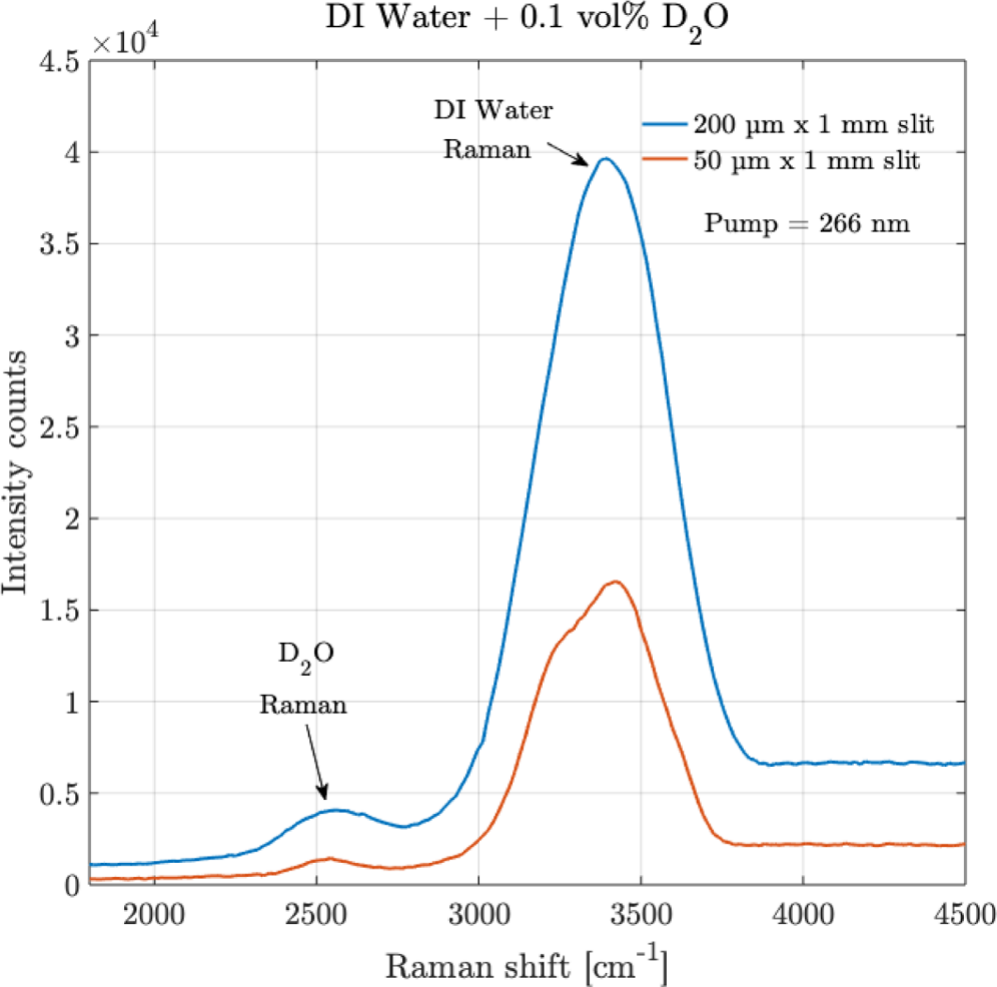
Raman measurements are shown for the isotope mixture of deuterium oxide (D_2_O) in DI water over 0.1 vol % of D_2_O, over two different slits of sizes 200 μm × 1 mm and 50 μm × 1 mm. Averaging = 10, integration time = 30 s. (c) Organic–inorganic mixture.

### 3.3 Organic-Inorganic Mixture

Different volumes % of IPA in DI water, of 50%, 30%, 10%, 1%, and 0.1%, were prepared and filled in UV quartz cuvettes and frozen. At first, as an initial measurement, as in the previous subsection, the detection measurements were performed over the 0.1 vol % IPA sample using the slit having the size of 200 μm × 1 mm. The measurements are shown in Figure 11. The Raman peak of DI water is detectable as before at 3421 cm^–1^. However, the Raman peak of IPA, which is expected to occur at 2900 cm^–1^, is not observable despite using the largest available slit size. However, when the measurement was made using a slit of a smaller size of 50 μm × 1 mm, while the intensity counts of the Raman spectrum reduced in Figure 11, the visibility of the Raman peak of IPA becomes clearer at 2900 cm^–1^, along with a more resolved Raman peak of DI water. This clear detection of the Raman peak of IPA arises due to the higher resolution (corresponding to 7 cm^–1^ wavenumber) provided by the smaller slit size compared to the larger slit size (corresponding to 20 cm^–1^ wavenumber). Qualitatively, this is because when the Raman peaks in a mixture are closer, the dominant Raman peak with higher intensity counts interferes and inundates the other peak, therefore hindering detection. A higher resolution (in this case, achieved with a smaller size slit) supports the presentation of distinct features in the Raman signal, which helps overcome the effect of the dominant close-by Raman peaks.

**Figure 11:**
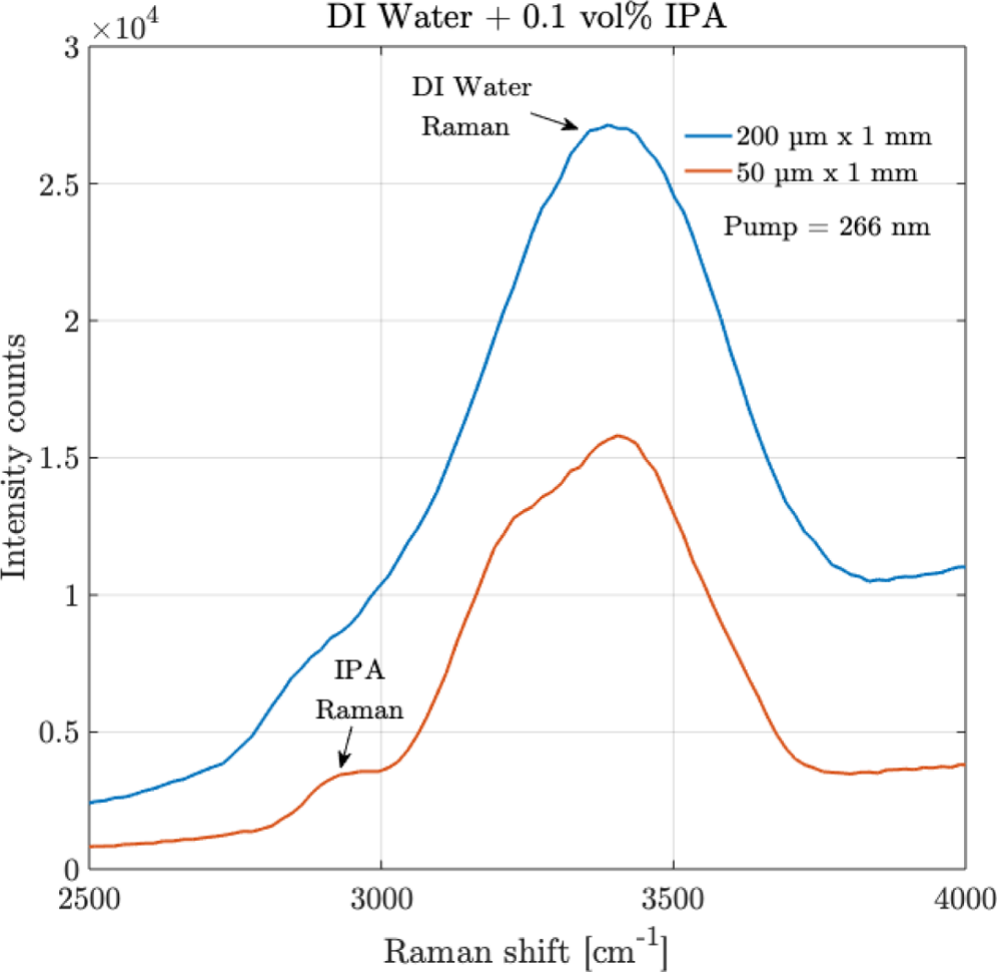
Raman measurements are shown for the organic–inorganic mixture of isopropyl alcohol (IPA) in DI water over 0.1 vol % of IPA over two different slits of sizes 200 μm × 1 mm and 50 μm × 1 mm. Averaging = 10, integration time = 30 s.

To further support the detection with the 50 μm × 1 mm slit, additional measurements were made using the sensor over different volume % of IPA. The results are shown in Figure 12. Following the measurements in Figure 12, using the 50 μm × 1 mm slit, the Raman peak of IPA at 2900 cm^–1^, is distinguishable from the Raman peak of DI water at 3421 cm^–1^., the volume % of IPA from 0.1 vol % to 50 vol % was investigated. These detection measurements, even at 0.1 vol % mixture, are possible due to the use of a deep-UV laser (providing a higher scattering) and the high quantum efficiency of 84% of the CCD in the spectrometer at 266 nm deep-UV wavelength, therefore, highlighting the deep-UV sensitive mixture detection measurement capabilities of the compact sensor.

**Figure 12:**
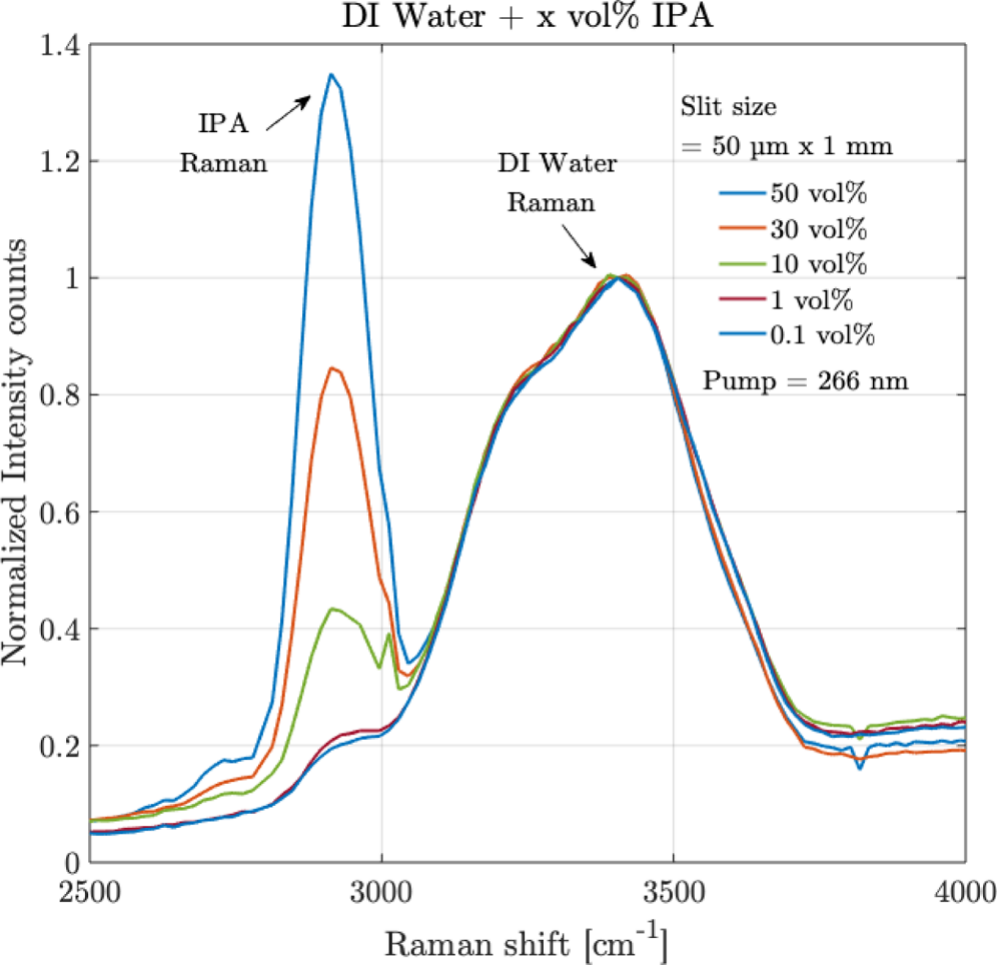
Raman measurements are shown for the organic–inorganic mixture of isopropyl alcohol (IPA) in DI water over different volumes % of IPA, 0.1%, 1%, 10%, 30%, and 50%. The *Y*-axis intensity counts are normalized concerning the Raman peak of DI water. Averaging = 10, integration time = 30 s, Slit size = 50 μm × 1 mm.

## 4 Conclusions

In summary, we have demonstrated the successful development and implementation of a compact, multifunctional chemical sensor that integrates deep-UV Raman-LIBS spectroscopic capabilities into a single hand-held unit. By employing a single 266 nm laser source coupled with an integrated autofocus mechanism, the sensor offers a streamlined and efficient alternative to conventional systems that rely on multiple laser wavelengths for Raman and LIBS functionalities. The deep-UV excitation enhances the Raman signal quality by reducing fluorescence interference observed with visible wavelengths, while also enabling effective plasma generation for LIBS. These benefits are evidenced by the ability of the sensor to detect target analytes in mixtures at concentrations as low as 0.1 vol % for both the isotopic water mixture (D_2_O/DI water) and the organic–inorganic mixture (IPA/DI water).

Our sensor’s design not only reduces the optical complexity typically associated with dual-laser systems but also leads to significant improvements in portability and operational simplicity. The compact sensor body, with a weight of only 38 g and dimensions of 70 × 60 × 40 mm^3^ (which can be held within the palm of an average human hand) combined with a lightweight laser and spectrometer, results in a total system weight of less than 500 g. This is particularly advantageous for in situ environmental detection applications and planetary exploration missions, where size, weight, and ease of use are critical parameters.

Our single-laser approach offers a competitive performance with the added benefits of reduced calibration complexity, lower cost, and enhanced system robustness. These features position the developed sensor as a promising tool for rapid, on-site diagnostics in environmental monitoring, food authentication, and space exploration.

Future work will focus on some promising avenues to further advance the technology of the compact chemical sensor. We plan to explore further miniaturization of the sensor by integrating on-board data processing and wireless communication capabilities, thereby enhancing real-time field analysis. Additionally, the development of comprehensive calibration libraries, coupled with advanced machine learning algorithms^11^ for automated signal interpretation, will be pursued. Also, conducting long-term robustness tests under various environmental conditions will validate its suitability for planetary exploration and other field applications, and possible commercialization.

## Data Availability

All data is available throughout the manuscript.
